# Optimized Sensitivity in Copper(II) Ion Detection: Sustainable Fabrication of Fluorescence Red-Shifted Graphene Quantum Dots via Electron-Withdrawing Modulation

**DOI:** 10.3390/molecules30061244

**Published:** 2025-03-10

**Authors:** Weitao Li, Qian Niu, Xinglong Pang, Shang Li, Yang Liu, Boyu Li, Shuangyan Li, Lei Wang, Huazhang Guo, Liang Wang

**Affiliations:** 1 Textile and Garment Industry of Research Institute, Zhongyuan University of Technology, Zhengzhou 450007, China; liweitao@zut.edu.cn (W.L.); 2022117681@zut.edu.cn (Q.N.); lishang@zut.edu.cn (S.L.); 2023117779@zut.edu.cn (Y.L.); boyu_li@126.com (B.L.); shuangyanli@126.com (S.L.); 2 Zhengzhou Key Laboratory of Smart Fabrics & Flexible Electronics Technology, Zhongyuan University of Technology, Zhengzhou 451191, China; 3 Institute of Nanochemistry and Nanobiology, School of Environmental and Chemical Engineering, Shanghai University, 99 Shangda Road, Shanghai 200444, China; pangxinglong@tsinghua-zj.edu.cn (X.P.); wangl@shu.edu.cn (L.W.); 4 Department of Environment, Yangtze Delta Region Institute of Tsinghua University, Jiaxing 314006, China

**Keywords:** graphene quantum dots (GQDs), electron-withdrawing modulation, copper detection, nitrogen doping, fluorescence red shift

## Abstract

Graphene quantum dots (GQDs) represent a class of promising nanomaterials characterized by adjustable optical properties, making them well suited for applications in biosensing and chemical detection. However, challenges persist in achieving scalable, cost-effective synthesis and enhancing detection sensitivity. In this study, we have developed a simple and environmentally friendly method to prepare blue graphene quantum dots, c-GQDs, using nitronaphthalene as a precursor, and yellow graphene quantum dots, y-GQDs, using nitronaphthalene doped acid. The quantum yield is 29.75%, and the average thickness is 2.08 nm and 3.95 nm, respectively. The synthesized c-GQDs exhibit a prominent cyan fluorescence at a wavelength of 490 nm under excitation at 380 nm, while the y-GQDs show a distinct yellow fluorescence at a wavelength of 540 nm under excitation at 494 nm. The introduction of p-aminobenzoic acid (PABA) introduced a marked red shift in fluorescence, attributed to the electron-withdrawing effect of the carboxyl groups on PABA. This key finding significantly enhanced the sensitivity of GQDs for detecting trace copper(II) ions (Cu^2+^), a heavy metal contaminant posing serious environmental risks. The fluorescence of the GQDs was selectively quenched in the presence of Cu^2+^, facilitating accurate and sensitive detection even in complex ion matrices. Mechanistic studies revealed that the quenching effect is driven by strong static quenching interactions, which inhibit non-radiative transitions. This work not only introduces a scalable method for producing high-performance GQDs but also highlights their potential as effective fluorescent probes for environmental monitoring and heavy metal ion detection.

## 1. Introduction

Graphene quantum dots (GQDs) have attracted considerable interest as a unique category of nanomaterials because of their outstanding optical characteristics, chemical stability, and biocompatibility, which position them as ideal candidates for diverse applications, including bio-sensing, drug delivery, and environmental monitoring [1,2]. Their unique attributes, such as size-dependent fluorescence, high quantum yield, and ease of functionalization, render them promising candidates for sensitive detection platforms [3,4]. However, achieving high sensitivity and selectivity in detecting specific analytes, particularly metal ions, remains a formidable challenge, which hinders their broader application in environmental and biological monitoring [5,6].

Copper(II) ions (Cu^2+^) are prevalent in both industrial processes and biological systems, playing critical roles in enzymatic catalysis and cellular metabolism [7,8]. While Cu^2+^ is essential for various physiological functions, elevated concentrations can result in severe environmental pollution and pose serious health risks, including disruptions in aquatic ecosystems and soil toxicity. Moreover, it has detrimental impacts on human health, including the induction of neurological disorders [9,10]. Thus, the establishment of dependable and highly sensitive methods for the detection of Cu^2+^ is of utmost importance for efficient environmental management and health risk evaluation. Traditional detection approaches for Cu^2+^, like atomic absorption spectroscopy, inductively coupled plasma mass spectrometry, and electrochemical sensors, offer high levels of accuracy and sensitivity [11,12]. However, these methods typically involve costly instrumentation, time-intensive sample preparation, and complex operational procedures, making them impractical for on-site or real-time monitoring applications [13,14]. Consequently, there is a growing demand for simpler, more cost-effective approaches that maintain high sensitivity and selectivity. Fluorescence-based probes have emerged as a promising alternative due to their operational simplicity, rapid response, and capability for real-time monitoring [15,16]. Among them, GQDs have shown great potential as fluorescent sensors owing to their tunable emission properties, excellent photostability, and versatile functionalization capacity. Nevertheless, the inherent challenge lies in achieving high specificity toward Cu^2+^ ions while minimizing interference from other coexisting metal ions in complex matrices [17,18].

**Scheme 1:**
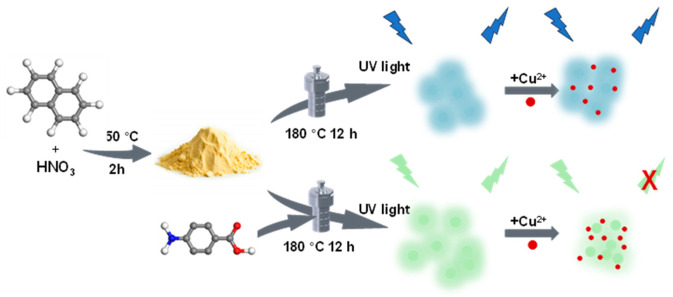
Preparation process diagram of graphene quantum dots.

In this study, we present an eco-friendly synthesis of nitrogen-doped GQDs from naphthalene, incorporating p-aminobenzoic acid (PABA) to leverage its electron-withdrawing effect (Scheme 1). The inclusion of PABA induces a distinct fluorescence red shift, significantly enhancing the sensitivity of the GQDs for Cu^2+^ detection [19]. By leveraging the electron-withdrawing effect of PABA, we aim to optimize the electronic environment of the GQDs, thereby developing a highly selective and sensitive fluorescent probe for Cu^2+^ ions [19,20]. This work not only establishes a sustainable and scalable synthetic strategy but also provides deeper mechanistic insights into the fluorescence modulation of GQDs, contributing to the advancement of GQD-based sensing technologies for heavy metal ion detection and environmental monitoring [21,22].

## 2. Results and Discussion

This study uses nitronaphthalene as the precursor and aminobenzoic acid as the modulator, employing a top–down hydrothermal synthesis process to prepare c-GQDs and y-GQDs. The preparation process for the two types of GQDs is shown in Scheme 1.

**Figure 1:**
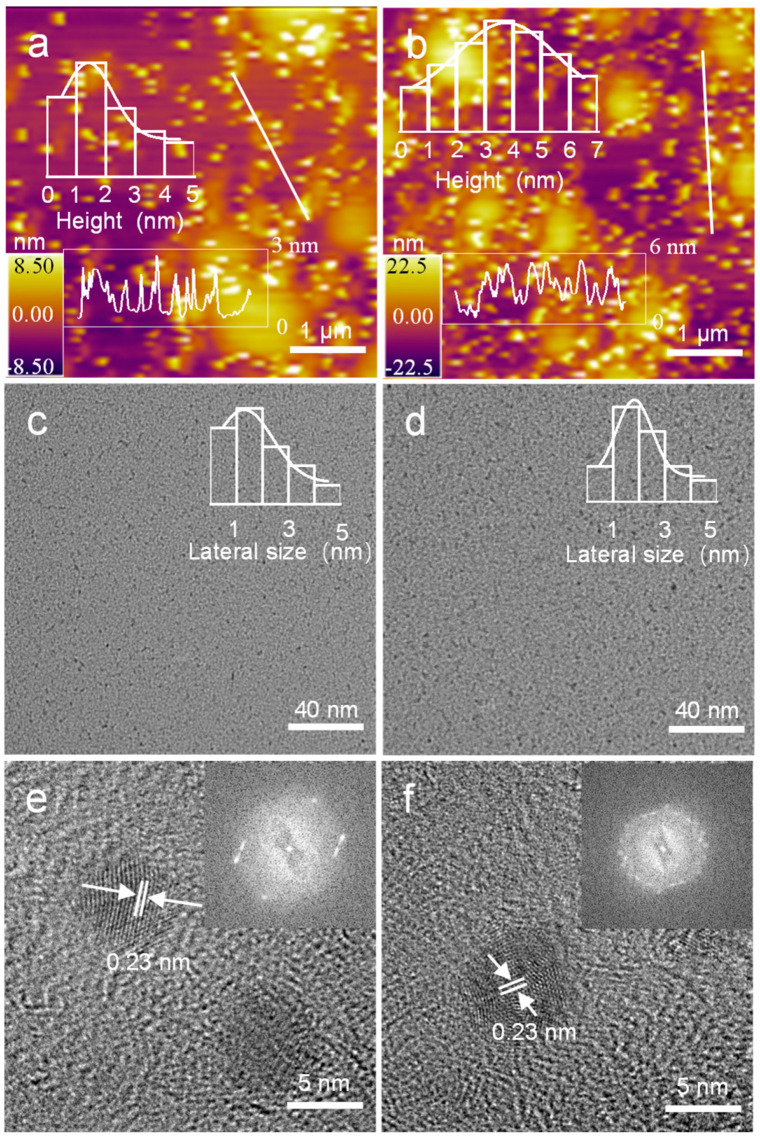
Schematic diagram of the morphology and structure of c-GQDs and y-GQDs. AFM and height distribution maps of c-GQDs (**a**) and y-GQDs (**b**). High-resolution TEM images of c-GQDs (**c**) and y-GQDs (**d**) (illustration: fast Fourier transform mode). TEM images and relative size distribution of c-GQDs (**e**) and y-GQDs (**f**).

The morphology of c-GQDs and y-GQDs is shown in Figure 1a,b. AFM height distribution histograms and TEM relative size distribution histograms were generated to characterize the tested samples. The atomic force microscopy (AFM) images and thickness distribution histograms of c-GQDs and y-GQDs are as follows: the average thickness of c-GQDs is 2.08 nm, and the average thickness of y-GQDs is 3.95 nm. This indicates that the graphene is composed of multiple layers, and the acidic modulation is favorable for the growth of the quantum dots. High-resolution transmission electron microscopy (HRTEM) images (Figure 1e,f) show that both c-GQDs and y-GQDs have a single crystalline structure with a lattice spacing of 0.23 nm, exhibiting good symmetry, similar to the benzene ring lattice structure of graphene. The TEM images and particle size distribution of the two types are shown in Figure 1c,d, indicating that the average particle size of c-GQDs is 1.8 nm and that of y-GQDs is 2.1 nm. The particle size distributions of both types are uniform.

**Figure 2:**
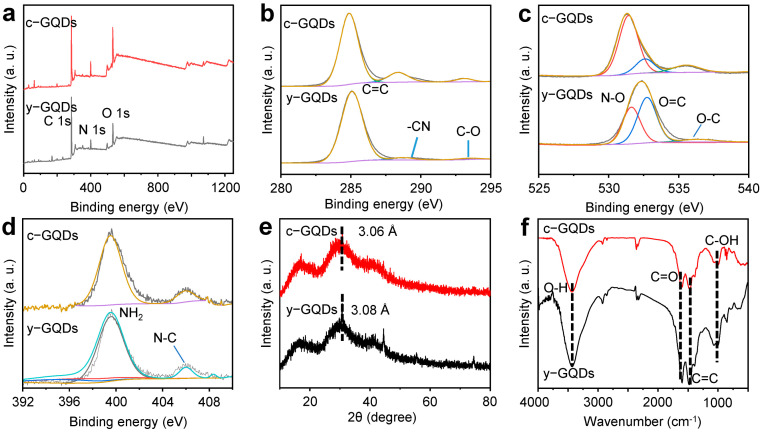
Structural characterization of GQDs. (**a**) XPS total spectrum of c-CQDs and y-GQDs. (**b**) C1s fine spectrum of c-CQDs and y-GQDs. (**c**) O1s fine spectrum of c-CQDs and y-GQDs. (**d**) N1s fine spectrum of c-CQDs and y-GQDs. (**e**) XRD pattern of c-GQDs and y-GQDs. (**f**) FT-IR spectra of c-GQDs and y-GQDs.

**Figure 3:**
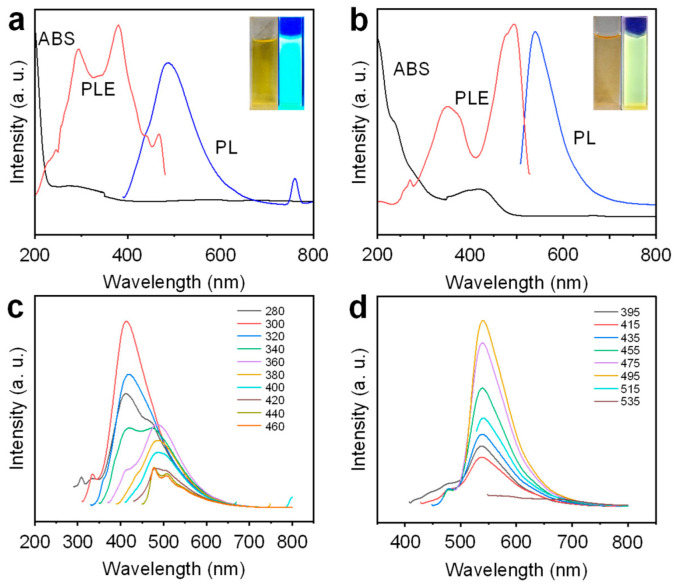
UV–visible absorption spectra and PL spectra of c-GQDs (**a**) and y-GQDs (**b**,**c**). Fluorescence spectra of c-GQDs at different excitation wavelengths (**d**). y-GQDs at different excitation wavelengths in the fluorescence spectrum.

XPS (X-ray photoelectron spectroscopy) analysis was carried out on the samples with the aim of ascertaining the surface composition of the GQDs. As presented in Figure 2a, the XPS full-spectrum profiles of c-GQDs and y-GQDs exhibit three distinct peaks. These peaks are attributed to C1s at a binding energy of 285 eV, N1s at 400 eV, and O1s at 532 eV, respectively. This indicates that the modification elements added after the incorporation of aminobenzoic acid remain unchanged, which is consistent with the Fourier transform infrared (FTIR) spectral data. Figure 3b–d show the high-resolution XPS spectra for C1s, N1s, and O1s of y-GQDs. The fine spectrum of C1s can be deconvoluted into three peaks: C-N (288.73 eV), C=C (285.28 eV), and C-O (293.48 eV). Similarly, the fine spectrum of O1s shows three peaks: N-O (532.13 eV), O=C (533.32 eV), and O-C (536.01 eV). Finally, the fine spectrum of N1s also shows three peaks: NH_2_ (399.58 eV) and N-C (408.08 eV). Compared to the full spectrum of y-GQDs, this reveals a significant reduction in nitrogen content and an increase in impurity peaks.

The XRD pattern of y-GQDs is shown in Figure 2e, exhibiting a prominent diffraction peak with an interlayer spacing of 3.08 Å. Additionally, c-GQDs show with an interlayer spacing of 3.06 Å (Figure 2e). The increased interlayer spacing observed in y-GQDs may be due to the modification with aminobenzoic acid [23,24]. In Figure 2f, the Fourier transform infrared (FTIR) spectrum of y-GQDs shows a prominent stretching vibration peak of the O-H bond at 3428 cm^−1^. The samples were thoroughly dried before infrared testing, indicating that the O-H bond originates from the y-GQDs, while peaks at 1603 cm^−1^, 1476 cm^−1^, and 1006 cm^−1^ correspond to C=O, C=C, and C-OH bonds, respectively. These functional groups suggest that the surface of y-GQDs contains a significant amount of amino and hydroxyl groups, indicating good hydrophilicity. The FTIR spectrum of c-GQDs displays similar functional groups to those of y-GQDs [25,26].

Figure S1 shows the Raman spectra of y-GQDs and c-GQDs. The typical D peak (1334 cm^−1^) and G peak (1560 cm^−1^) of GQDs were observed, corresponding to the disordered sp^3^ hybrid state (D peak) and ordered sp^2^ state (G peak). From Figure S2b (Supplementary Materials), it can be seen that the D peak of c-GQDs appears at 1347 cm^−1^ and the G peak appears at 1550 cm^−1^. The _ID_/_IG_ values of y-GQDs are higher than those of c-GQDs [27,28]. This may be due to the introduction of more structural defects, such as vacancies, edge defects, etc., in the acid-modified y-GQD, resulting in an enhancement of the D peak. At the same time, the introduction of defects also leads to an enhancement of localized sp^2^ localization, thereby affecting the intensity of the G peak. Meanwhile, doping with acid may alter the electronic structure of GQDs by introducing heteroatoms, thereby affecting the intensity and position of the D and G peaks. These results indicate that the graphitization degree of y-GQDs is lower than that of c-GQDs.

The XPS results of c-GQDs and y-GQDs indicate that both contain strong electron-donating amino groups (NH_2_). We infer that NH_2_ is covered by O-H bonds, which are weak electron-withdrawing carboxyl groups (−COOH), suggesting the presence of moderately electron-withdrawing groups [29]. Based on the XPS analysis of the ratio of electron-withdrawing and electron-donating groups in GQDs, it is observed that during the fluorescence tuning process from blue to yellow, the ratio of electron donating groups (NH_2_) to carbon atoms further decreases. Table S1 shows the proportion of elements, where an increasing proportion of C=O indicates an increasing proportion of the electron-withdrawing −COOH group in GQDs [30,31], suggesting that the increase in electron-withdrawing groups leads to a red shift in the fluorescence of GQDs.

### 2.1. Characterization of Optical Properties of GQDs

The UV absorption and photoluminescence (PL) spectra of the two types of GQDs are shown in the figure. The maximum absorption peaks of c-GQDs and y-GQDs appear at 380 nm and 494 nm, respectively, which are attributed to the π-π* transition of conjugated carbon–carbon double bonds. Under natural light, both c-GQDs and y-GQDs appear yellow; however, under ultraviolet light, c-GQDs emit blue fluorescence, while y-GQDs emit yellow fluorescence, indicating that GQDs exhibit fluorescence after the hydrothermal reaction [32,33]. The optimal excitation wavelength for c-GQDs is 360 nm, with an emission wavelength of 490 nm, while the optimal excitation wavelength for y-GQDs is 495 nm, with an emission wavelength of 540 nm [34]. Figure 3c,d show that, due to the highly ordered graphite structure of both types of GQDs, the peak intensity changes with varying excitation wavelengths, resulting in different peak positions in the PL spectra, with no deviation in the excitation.

The thermal stability of the two types of GQDs was evaluated through a series of tests. First, the fluorescence intensity of 3 mL of the original GQD solution was measured. Then, the GQD solution was dried and dispersed in an equal volume of deionized water, after which the fluorescence intensity was measured again. As shown in Figure S2, compared to regular GQDs, the nitrogen-doped y-GQDs exhibit higher thermal stability. Over a period of two months, fluorescence tests were continuously conducted on the c-GQD and y-GQD solutions for several days, and the resulting data were compared with the initial measurements. The results indicated in Figure S3 suggest that the fluorescence intensity of both c-GQDs and y-GQDs changed very little, indicating that they have excellent temporal stability.

### 2.2. GQDs for Ion Detection

**Figure 4:**
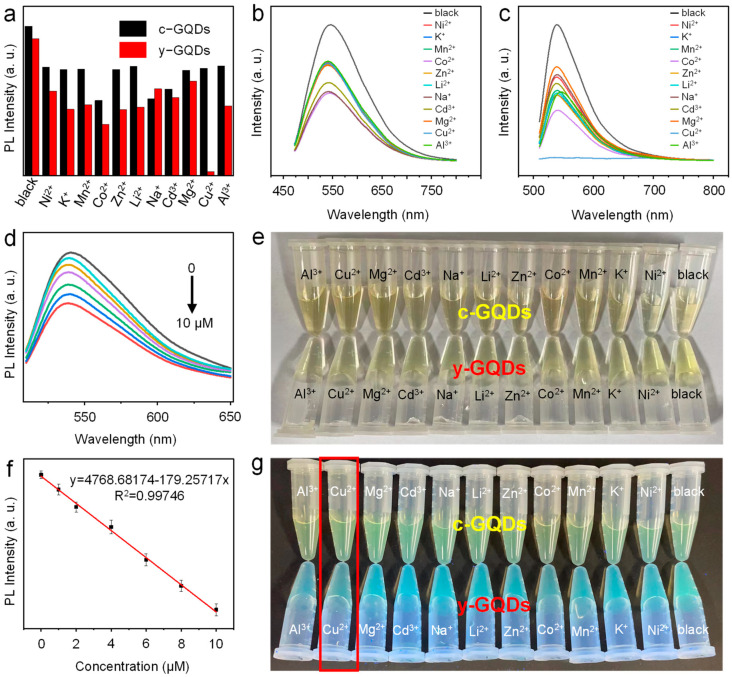
(**a**) Fluorescence spectrum intensity maps of different ion solutions and blank solutions. (**b**) Fluorescence spectra of c-GQDs containing different metal ions. (**c**) Fluorescence spectra of y-GQDs containing different metal ions. (**d**) Fluorescence spectra of y-GQDs at different ion concentrations. Photos of c-GQDs and y-GQDs containing solutions of different metal ions under natural light (**e**) and ultraviolet light (**g**). (**f**) Cu^2+^ concentration is between 0 and 10 μM. A linear relationship is seen between y-GQDs and PL intensity at different concentrations.

In this experiment, the selective recognition ability of GQDs for metal ions was tested using various metal ions. A 10 mol/L metal ion solution was prepared and added to the c-GQD and y-GQD solutions to detect their fluorescence intensity. The observed fluorescence quenching results indicate that after the addition of different metal ions, the fluorescence intensity of the GQDs decreases uniformly, but the decrease is not significant, as shown in Figure 4a–c. Additionally, Figure 4b shows that the fluorescence quenching of c-GQDs is minimal, indicating that they are not suitable for detecting these ions. Furthermore, Figure 4g shows that after the addition of metal ion solutions, especially Cu^2+^, the fluorescence intensity of y-GQDs significantly decreases. The data in Figure 4c further confirm this, showing that Cu^2+^ has the greatest effect on fluorescence intensity compared to other metal ion solutions. Notably, as seen in Figure 4a, Cu^2+^ causes the most significant fluorescence quenching effect among all the metal ions, while the effect of other ions on y-GQDs is relatively less pronounced.

The fluorescence intensity of y-GQDs was measured as the Cu^2+^ concentration varied from 0 μM to 10 μM, and the phenomenon of its linear relationship with Cu^2+^ concentration was probed. As depicted in Figure 4d, when the concentration of Cu^2+^ ions rose from 0 μM to 10 μM, the fluorescence intensity of the y-GQD solution progressively declined [35,36]. Additionally, as can be observed in Figure 4f, a robust linear correlation exists between the concentration of Cu^2+^ ions and the PL (photoluminescence) fluorescence intensity of y-GQDs.

**Figure 5:**
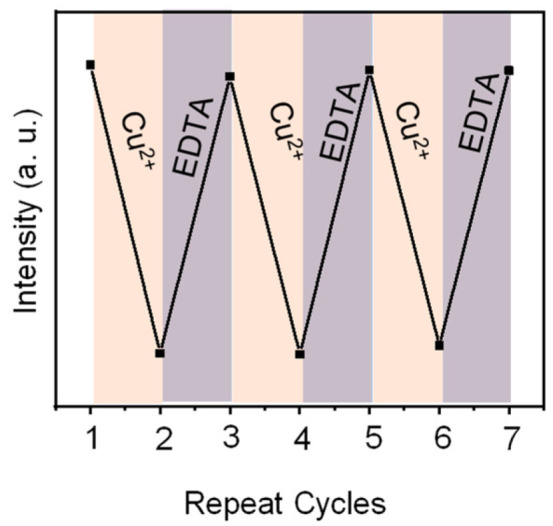
The ion reduction process of y-GQDs after adding EDTA.

The correlation coefficient (R^2^) is 0.99746. Subsequently, the fluorescence intensity of y-GQDs was measured in the presence of EDTA and Cu^2+^ to determine their cycling activity. As shown in Figure 5, EDTA can bleach the emission band of the Cu^2+^ signal. EDTA removes it from the surface of GQDs by binding with metal ions. By adding a certain amount of EDTA to the GQD solution, mixed with an ionic solution, the fluorescence intensity can be roughly restored.

To verify the practicality of y-GQDs in environmental testing, we separately added pesticides (Bordeaux mixture) containing copper ions and dye wastewater containing copper salts to y-GQDs. Fluorescence tests were conducted on them separately, as shown in Figure S4. The Cu^2+^ concentration in the obtained pesticide was 75 mmol/L, and the copper ion concentration in the dye wastewater was 1.5 mmol/L. The results indicate that y-GQDs have sensitive and efficient detection capabilities for Cu^2+^.

**Figure 6:**
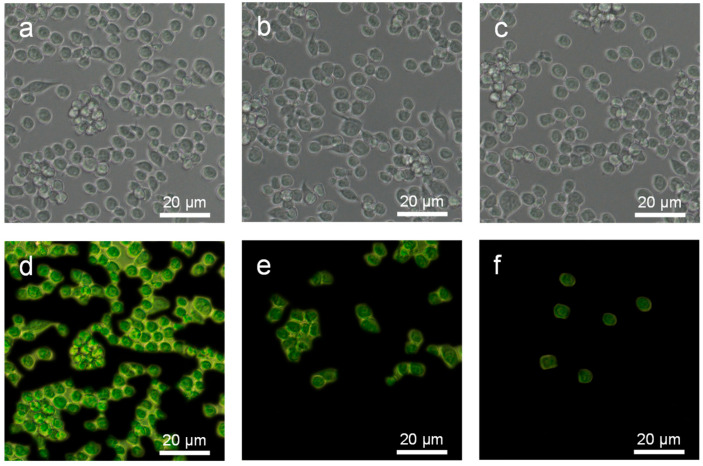
Cell imaging, bright field, and fluorescence images of y-GQDs: (**a**) and (**d**) without added Cu^2+^; (**b**) and (**e**) with 1 μM/mL Cu^2+^; (**c**) and (**f**) with 5 μM/mL Cu^2+^.

To investigate the application of y-GQDs in biological detection, the effect of Cu^2+^ on cellular imaging was evaluated. Mouse cells were incubated with Cu^2+^ (1 μM/mL and 5 μM/mL) and subsequently treated with y-GQDs. Compared to the control group (Figure 6a,d), a pronounced concentration-dependent quenching of y-GQD fluorescence was observed in Cu^2+^-treated cells (Figure 6b,c,e,f). Notably, fluorescence was nearly completely quenched at higher Cu^2+^ concentrations, demonstrating the potential of y-GQDs as a sensitive probe for Cu^2+^ detection in biological systems.

## 3. Experimental Section

### 3.1. Materials

All chemicals used in this experiment were of analytical grade and used without further purification. Naphthalene, p-aminobenzoic acid (PABA), concentrated nitric acid, and the metal ion-related drugs were purchased from Sinopharm Chemical Reagent Co., Ltd. The metal ion solutions (Cu^2+^, Mn^2+^, Co^2+^, Cd^2+^, K^+^, Al^3+^, Na^+^, and Ni^2+^) were prepared using metal salts dissolved in deionized water. For all experiments, deionized water (18.2 MΩ·cm) was used.

### 3.2. Instrumentation and Characterizations

An array of advanced instruments were employed for the characterization and analysis of the synthesized GQDs. The fluorescent properties of GQDs were studied under high-intensity UV light (FC-100/FA, Spectroline, Melville, NY, USA). Raman spectral analysis was conducted using a confocal micro-Raman spectrometer (X-plora PLUS, Shanghai, China), and the height profiles of the GQDs were measured with an atomic force microscope (AFM) (MFP-3D Infinity, Oxford, UK). The infrared spectrum of the GQDs was recorded using a Fourier transform infrared (FT-IR) machine (ALPHA II, Bruker, Germany), and X-ray diffraction (XRD) patterns were obtained using a multi-purpose X-ray diffractometer (D2 PHASER, Bruker, Germany).

Fluorescence intensity was measured using a fluorescence spectrophotometer (RF-6000, Shimadzu Corporation, Kyoto, Japan), while high-resolution transmission electron microscopy (TEM) images were acquired using a TEM (JEOL JEM-F200, Japan Electronics Co., Ltd., Tokyo, Japan). The morphologies of GQDs and GQDs/MLB were obtained by scanning electron microscopy (SEM) (ZEISS Sigma 360, Jena, Germany). UV-Vis absorption spectra were recorded with a spectrophotometer (LAMBDA750, PerkinElmer, Waltham, MA, USA). X-ray photoelectron spectroscopy (XPS) analysis was conducted using an X-ray diffractometer (K-Alpha, Cambridge, UK) to obtain atomic composition data. Fluorescence lifetime maps were obtained using a steady-state/transient fluorescence spectrometer (Edinburgh FLS980, Edinburgh, UK).

### 3.3. Synthesis of c-GQDs and y-GQDs

Preparation of nitronaphthalene: A total volume of 27 mL of nitric acid was added into a three-necked flask, and then 50 mmol of naphthalene was added at room temperature. The reaction mixture was heated to 50 °C and stirred magnetically for 2 h. The product was filtered at room temperature, washed three times with nitric acid, and dissolved in hot ethanol. Activated carbon was then added for decolorization. The mixture was filtered while hot, and yellow powder nitronaphthalene was precipitated after cooling.

Preparation of blue c-GQDs: c-GQDs were synthesized by the hydrothermal method. A total of 0.1 g of nitronaphthalene was weighed and then 9 mL of deionized water was added to the nitronaphthalene. The nitronaphthalene was ultrasonically shaken until it fully dissolved in the deionized water. Then, the suspension was transferred to a high-pressure reactor (10 mL) lined with polytetrafluoroethylene that had been previously cleaned with nitric acid. The reactor was heated to 180 °C in an oven and reacted for 12 h. After that, it was naturally cooled to room temperature. The product was filtered through a 0.22 μM and 25 nm microporous membrane to remove insoluble impurities. By irradiating the obtained product with ultraviolet light, we concluded that we successfully prepared the blue GQD solution.

Preparation of yellow y-GQDs: y-GQDs were synthesized by the hydrothermal method. A total of 0.1 g of nitronaphthalene and 0.1 g of p-aminobenzoic acid were weighed and mixed. Then, 9 mL of deionized water was added to the mixture. The mixture was ultrasonically shaken until it fully dissolved in the deionized water. Then, the suspension was transferred to a high-pressure reactor (10 mL) lined with polytetrafluoroethylene that had been previously cleaned with nitric acid. The reactor was heated to 180 °C in an oven and reacted for 12 h. After that, it was naturally cooled to room temperature. The product was filtered through a 0.22 μM and 25 nm microporous membrane to remove insoluble impurities. By irradiating the obtained product with ultraviolet light, we concluded that we successfully prepared the yellow GQD solution.

### 3.4. Metal Ion Detection

We selected some metal ions (Ni^2+^, K^+^, Mn^2+^, Co^2+^, Zn^2+^, Li^2+^, Na^+^, Cd^2+^, Mg^2+^, Cu^2+^, and Al^3+^) for this experiment to detect the effect of metal ions on the fluorescence intensity of c-GQDs and y-GQDs [37,38]. We uniformly adjusted the concentration of the metal ion solution to 10 mM. Then, a certain amount of metal ion solution was mixed with c-GQDs and y-GQDs to test the fluorescence quenching degree of the quantum dot solution after adding different metal ions [39,40].

## 4. Conclusions

This study synthesized yellow fluorescent y-GQDs using nitronaphthalene as a precursor and aminobenzoic acid as a modifier, through a bottom–up solvothermal method. The synthesized y-GQDs exhibited excellent fluorescence stability, significant excitation wavelength dependence, and a remarkable quantum yield of up to 29.75%. The optical properties, morphology, and structure of the synthesized y-GQDs were characterized. The results show that the prepared y-GQDs exhibit excellent performance and possess the characteristics of graphene quantum dots. Additionally, the selective recognition ability of y-GQDs for metal ions was tested. Under the influence of Cu^2+^, the fluorescence quenching activity of y-GQDs was more pronounced, indicating that y-GQDs have the ability to detect Cu^2+^. The application of y-GQDs in chemical sensing will become increasingly widespread, as the interaction between GQDs and metal ions is usually completed in a short period of time, making it suitable for real-time detection and rapid analysis. Moreover, GQDs have low toxicity and good biocompatibility, making them suitable for detecting metal ions in biological and environmental samples. The preparation of raw materials is abundant, the cost is low, and it is suitable for large-scale applications. GQDs exhibit high sensitivity, selectivity, rapid response, and good biocompatibility in metal ion detection, making them a highly promising detection material.

## Data Availability

Data will be made available on request due to privacy.

## Supplementary Materials

The following supporting information can be downloaded at https://www.mdpi.com/article/10.3390/molecules30061244/s1, Figure S1. Raman spectra of y-GQDs (a) and c-GQDs (b); Figure S2. Fluorescence spectra of y-GQDs (a) and c-GQDs (b) before and after thermal stability; Figure S3. Bar graph of fluorescence peak intensity of y-GQDs (a) and c-GQDs (b) at different times; Figure S4. Application of y-GQDs in Environmental Monitoring; Table S1. XPS measures the element ratios of c-GQDs and y-GQDs in the spectra.
