# Supplementary material for: Optimized Sensitivity in Copper(II) Ion Detection: Sustainable Fabrication of Fluorescence Red-Shifted Graphene Quantum Dots via Electron-Withdrawing Modulation
Source: Molecules. 2025 Mar 10;30(6):1244. doi: 10.3390/molecules30061244 (PMC11946535; doi:10.3390/molecules30061244)
Supplement: Supplementary file 1 [file molecules-30-01244-s001.zip › molecules-3430538-supplementary.pdf]

Supporting Information

Optimized Sensitivity in Copper(II) Ion Detection: Sustainable Fabrication of Fluorescence Red-Shifted Graphene Quantum Dots via Electron-Withdrawing Modulation

Weitao Li [1,2], Qian Niu [1,2], Xinglong Pang [3,4], Shang Li [1,2], Yang Liu [1,2], Boyu Li [1,2], Shuangyan Li [1], Lei Wang [1,*], Huazhang Guo [3,*] and Liang Wang [3]

[1] Textile and Garment Industry of Research Institute, Zhongyuan University of Technology,
Zhengzhou 450007, China; liweitao@zut.edu.cn (W.L.); 2022117681@zut.edu.cn (Q.N.);
lishang@zut.edu.cn (S.L.); 2023117779@zut.edu.cn (Y.L.); boyu_li@126.com (B.L.);
shuangyanli@126.com (S.L.)

[2] Zhengzhou Key Laboratory of Smart Fabrics & Flexible Electronics Technology, Zhongyuan University of Technology, Zhengzhou 451191, China

[3] Institute of Nanochemistry and Nanobiology, School of Environmental and Chemical Engineering,
Shanghai University, 99 Shangda Road, Shanghai 200444, China; pangxinglong@tsinghua-zj.edu.cn (X.P.); wangl@shu.edu.cn (L.W.)

[4] Department of Environment, Yangtze Delta Region Institute of Tsinghua University, Jiaxing 314006, China

* Correspondence: wanglei@zut.edu.cn (L.W.); guohuazhang@shu.edu.cn (H.G.)

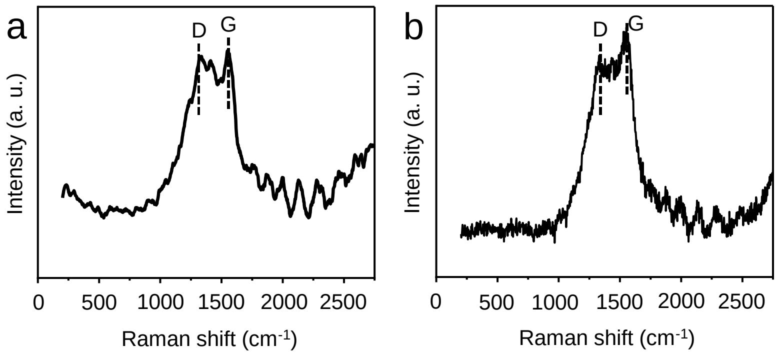

Figure S1. Raman spectra of y-GQDs (a) and c-GQDs (b).

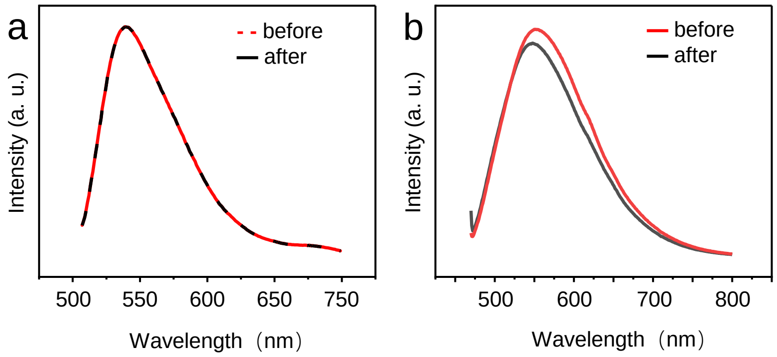

Figure S2. Fluorescence spectra of y-GQDs (a) and c-GQDs (b) before and after thermal stability.

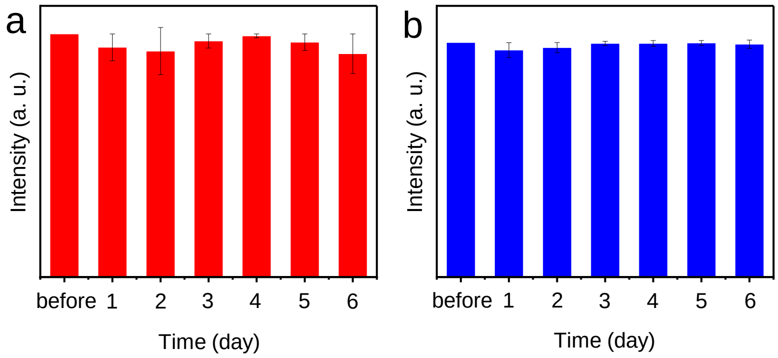

Figure S3. Bar graph of fluorescence peak intensity of y-GQDs (a) and c-GQDs (b) at different times.

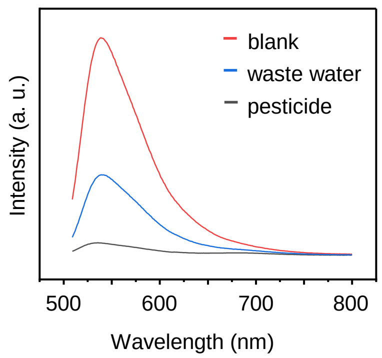

Figure S4. Application of y-GQDs in Environmental Monitoring.

Table S1. XPS measures the element ratios of c-GQDs and y-GQDs in the spectra.

**Element**	**C**	**N**	**O**
c-GQDs	65.73%	5.82%	28.45%
y-GQDs	70.45%	4.58%	24.97%
